# Singular Case of Foreign Substances in the Intestinal Canal

**Published:** 1853-06

**Authors:** D. Hayes Agnew

**Affiliations:** Philadelphia


### *Singular Case of Foreign Substances in the Intestinal Canal.*

By D. Hayes Agnew, M. D., Philadelphia.

The following case I am induced to report from its very singular character:—On examining the body of an individual who, I believe, labored under some mental alienation during life, my attention was attracted to an adhesion between the parietal and visceral layer of peritoneum over the cœcum, upon the separation of which a small opening was perceived through the walls of the intestine, disclosing a dark-looking substance, which, upon examination, proved to be a large mass of straw, little less than an ordinary sized fist, and firmly impacted in all the space below the ileo-cœcal valve. Noticing the transverse colon very much distended, an incision was made into its cavity, where were found a pair of suspenders, three rollers, and a quantity of thread, interwoven with one another. The webbing, which evidently was his suspenders, exceeded one and a quarter inches in breadth, and must be several feet in length, inasmuch as it extended through the ascending, transverse, and a portion of the descending colon, and doubled in several places upon itself. The rollers were of ordinary muslin, over one inch in width and the same in diameter, but which must have been of much greater size when swallowed, as they had, in their progress along the intestines, become unrolled, leaving long ends which were encased within layers of fæculent matter. The peritonitis, which no doubt had been the principal cause of death, was not, however, produced by the escape of any intestinal matter into the serous cavity, no such discharge having occurred. The opening into the cœcum only presented itself after the reflected layer of the peritoneum was separated therefrom. Had life been prolonged, it is highly probable that the ulceration would have extended through the walls of the abdomen, and the cœcal contents passed out by this artificial route.

